# Supplementary material for: Exploring bacterial diversity via a curated and searchable snapshot of archived DNA sequences
Source: PLoS Biol. 2021 Nov 9;19(11):e3001421. doi: 10.1371/journal.pbio.3001421 (PMC8577725; doi:10.1371/journal.pbio.3001421)
Supplement: S1 Table — (DOCX) [file pbio.3001421.s001.docx]

S1 Table. Projects under Umbrella project PRJEB46036

| **Project accession** | **Species** | **Number of assemblies** |
| --- | --- | --- |
| PRJEB45962 | *S. enterica* | 181871 |
| PRJEB45963 | *Escherichia* and *Shigella* | 90364 |
| PRJEB45964 | *S. pneumoniae* | 51517 |
| PRJEB45965 | *M. tuberculosis* | 48727 |
| PRJEB45966 | *S. aureus* | 48418 |
| PRJEB45967 | *C. jejuni* | 28498 |
| PRJEB45968 | *L. monocytogenes* | 24940 |
| PRJEB45969 | *N. memingitidis* | 17306 |
| PRJEB45970 | *S. pyogenes* | 16830 |
| PRJEB45971 | *C. difficile* | 13713 |
| PRJEB45972 | *K. pneumoniae* | 13621 |
| PRJEB45973 | *S. agalactiae* | 10302 |
| PRJEB45974 | *C. coli* | 8978 |
| PRJEB45975 | *N. gonorrhoeae* | 8898 |
| PRJEB45976 | *E. faecium* | 8635 |
| PRJEB45977 | *P. aeruginosa* | 6371 |
| PRJEB45978 | *V. cholerae* | 5634 |
| PRJEB45979 | *A. baumannii* | 5162 |
| PRJEB45980 | *M. abscessus* | 2707 |
| PRJEB45981 | *L. pneumophila* | 2296 |
| PRJEB45982 | Other species | 66617 |
