# Supplementary figures and images for: Exploring bacterial diversity via a curated and searchable snapshot of archived DNA sequences
Source: PLoS Biol. 2021 Nov 9;19(11):e3001421. doi: 10.1371/journal.pbio.3001421 (PMC8577725; doi:10.1371/journal.pbio.3001421)

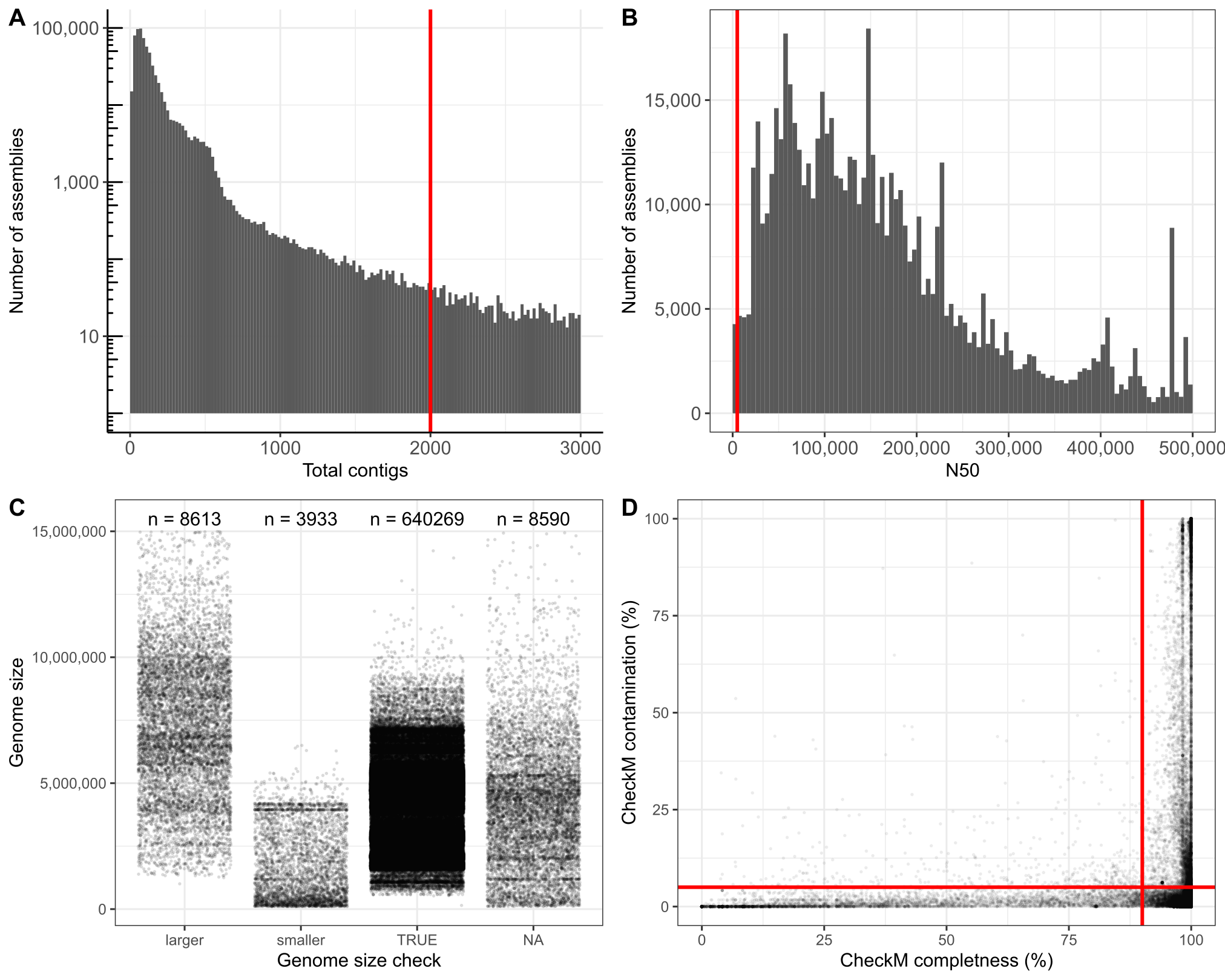

Supplement: S1 Fig — (A) Distribution of number of contigs per assembly in the collection. A total of 1,766 assemblies had greater than 3,000 contigs. Red line: Assemblies with more than 2,000 contigs were filtered from the high-quality assemblies. (B) Distribution of the N50 of each of the assemblies in the collection and 26,142 had an N50 of greater than 500,000. Red line: assemblies with an N50 less then 5,000 were filtered from the high-quality assemblies. (C) Comparison of genome size of each assembly to that expected of its species. Where available, the genome size range accepted for each species was extracted from ftp://ftp.ncbi.nlm.nih.gov/genomes/ASSEMBLY_REPORTS/species_genome_size.txt.gz, downloaded August 27, 2020. Those genomes that are greater than or less than the expected length was filtered from the high-quality assemblies. (D) Correlation between the genome completeness and contamination percentages produced by CheckM for each assembly. A total of 1,785 assemblies had a contamination score greater then 100%. Red lines indicate cutoffs applied; bottom right corner are the high-quality genomes. The data underlying this figure may be found in https://doi.org/10.6084/m9.figshare.16437939. (TIFF) [file pbio.3001421.s002.tiff]

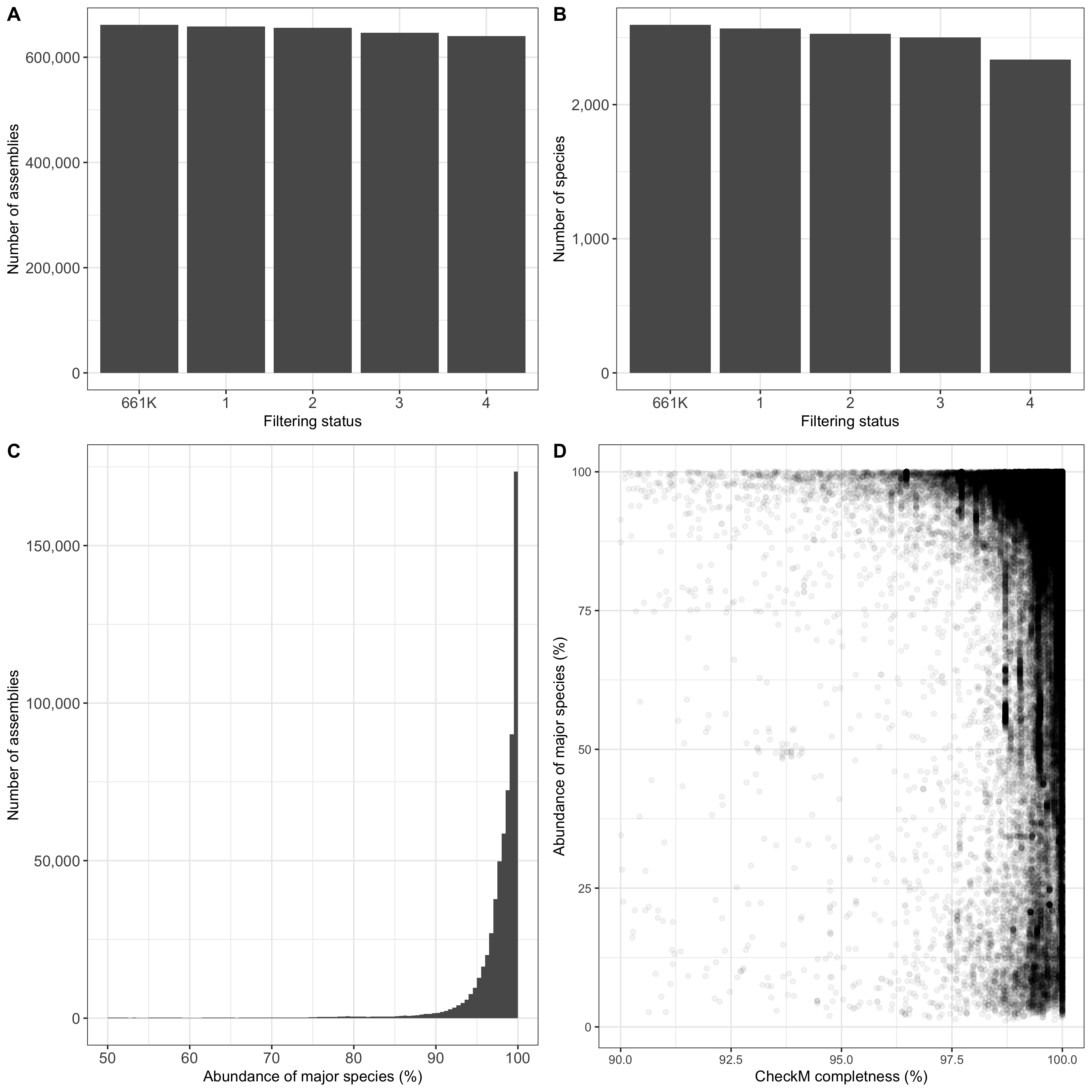

Supplement: S2 Fig — Number of (A) assemblies and (B) species remaining following each stage of filtering. Status 1; removal of genomes with >2,000 contigs, status 2; removal of genomes with an N50 <5,000, status 3; removal of genomes with length outside the range expected for that species (note: if expected range is not known, the assemblies are kept), status 4; assemblies with a completeness score = >90% and with a contamination score = <5%. A total of 639,981 assemblies passed the 4 levels of filtering and are the high-quality genomes. (C) Distribution of high-quality assemblies (filtering status 4) with >50% abundance of major species. A total of 9,595 assemblies were below this threshold. (D) Within-sample abundance of major species vs completeness of the high-quality assemblies. For (C) and (D), the abundance of major species is the adjusted abundance values (see Methods). The data underlying this figure may be found in https://doi.org/10.6084/m9.figshare.16437939. (TIFF) [file pbio.3001421.s003.tiff]

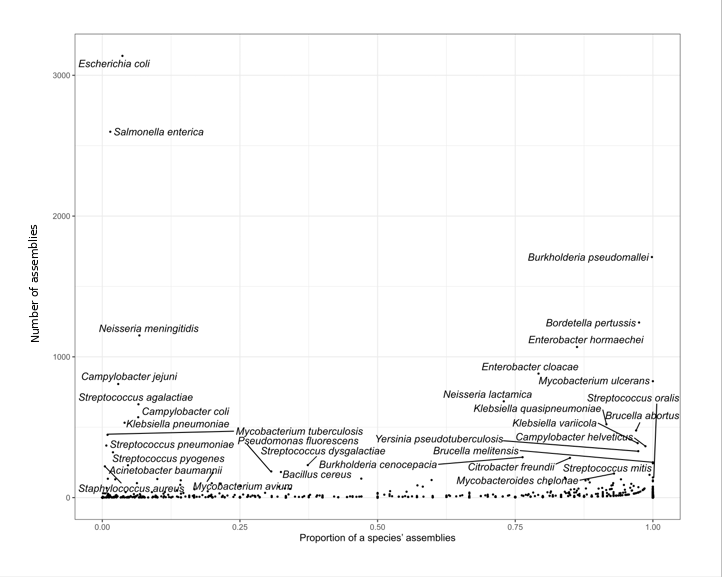

Supplement: S3 Fig — The data underlying this figure may be found in https://doi.org/10.6084/m9.figshare.16437939. (TIFF) [file pbio.3001421.s004.tiff]

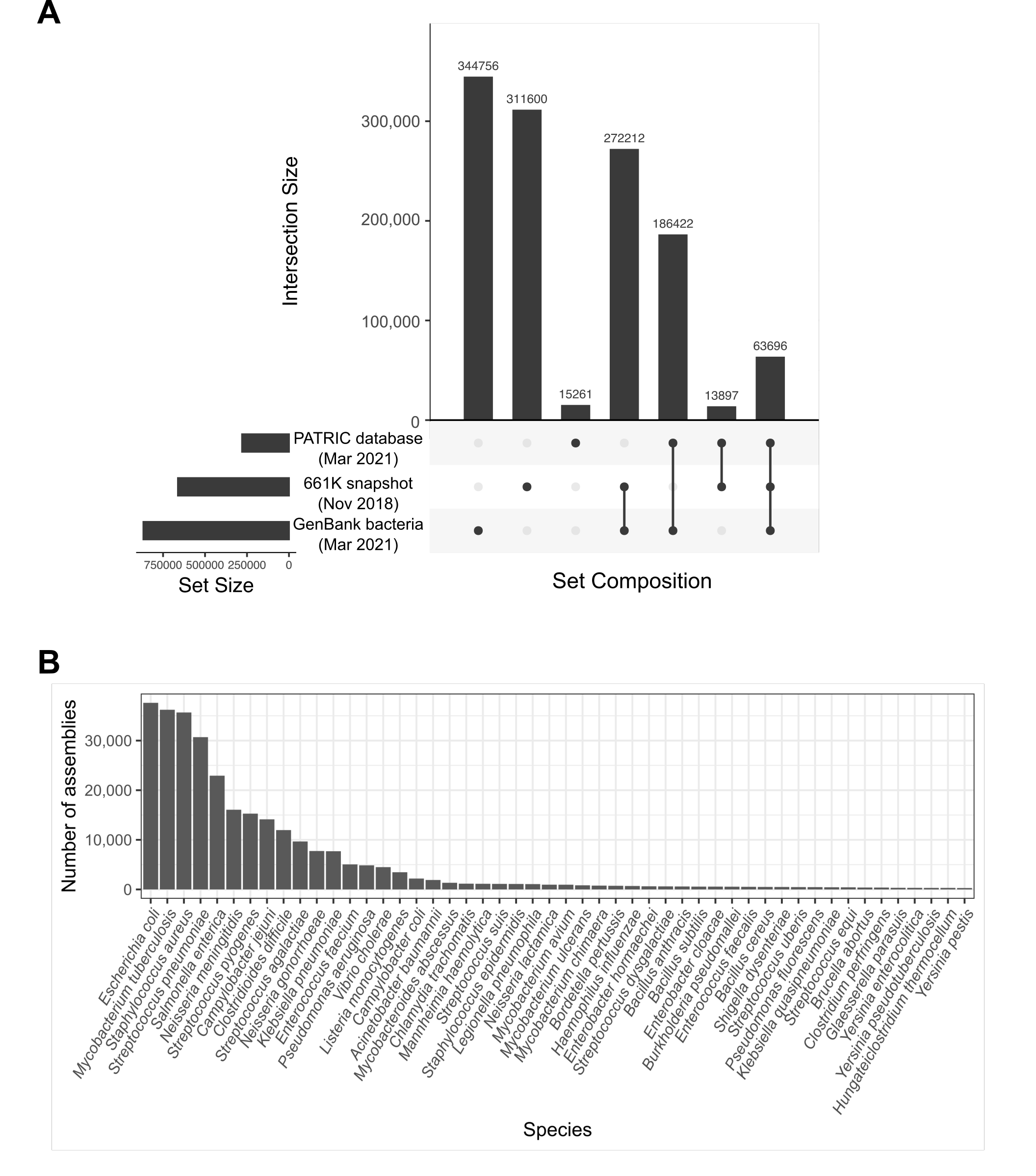

Supplement: S4 Fig — (A) Upset plot shows the number of shared sample accessions (also called biosample) in the 661K snapshot, the GenBank bacterial, and PATRIC databases. Each column corresponds to an exclusive intersection that includes the elements denoted by the dark circles, but not of the others. (B) The top 50 species in the 311,600 sample accessions unique to the 661K snapshot. The data underlying this figure may be found in https://doi.org/10.6084/m9.figshare.16437939. (TIFF) [file pbio.3001421.s005.tiff]

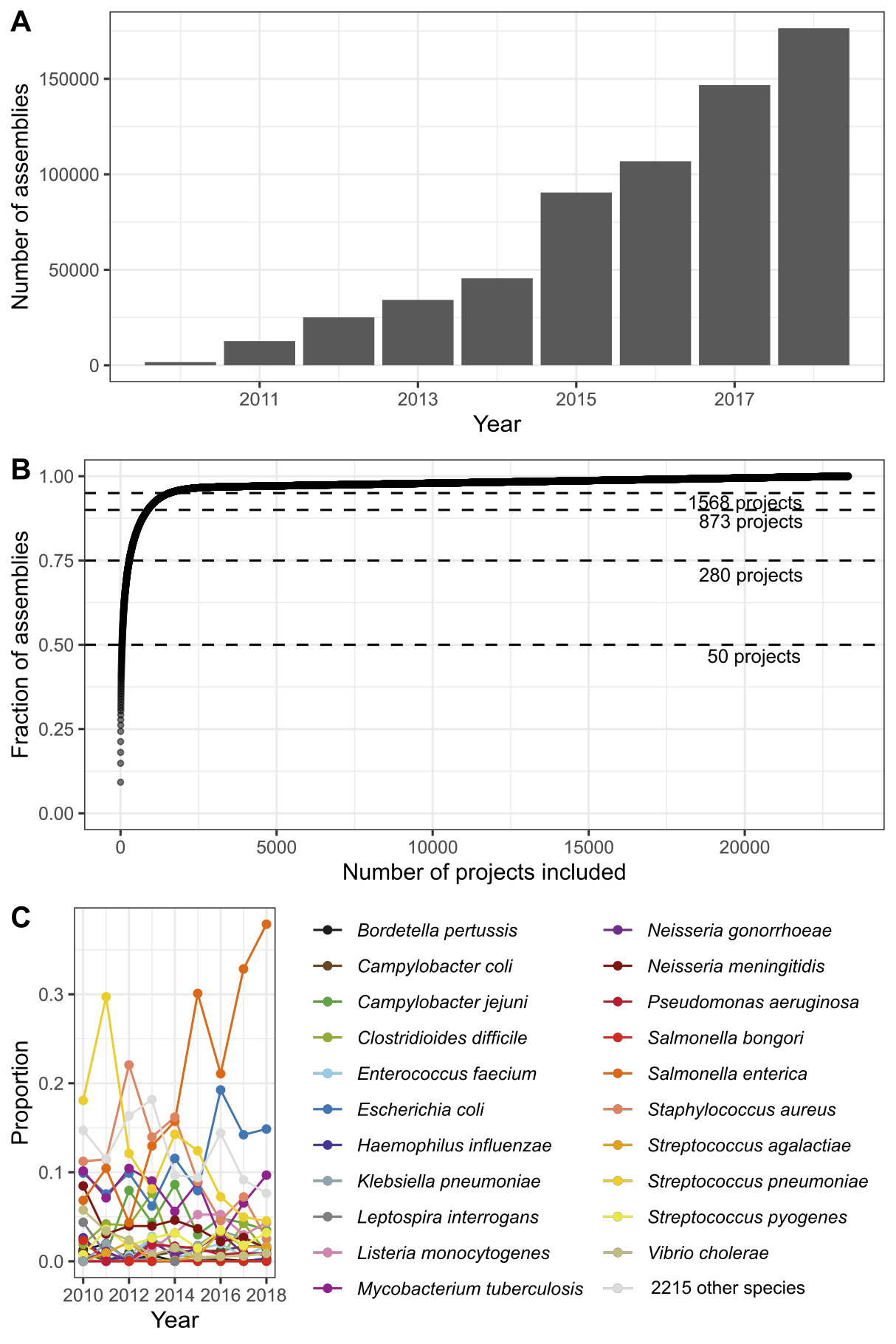

Supplement: S5 Fig — (A) Breakdown of assemblies by year first public in the ENA. (B) Fraction of assemblies covered by accumulating projects. (C) Tracking proportions of the top 10 bacterial species for a year. The data underlying this figure may be found in https://doi.org/10.6084/m9.figshare.16437939. ENA, European Nucleotide Archive. (TIFF) [file pbio.3001421.s006.tiff]

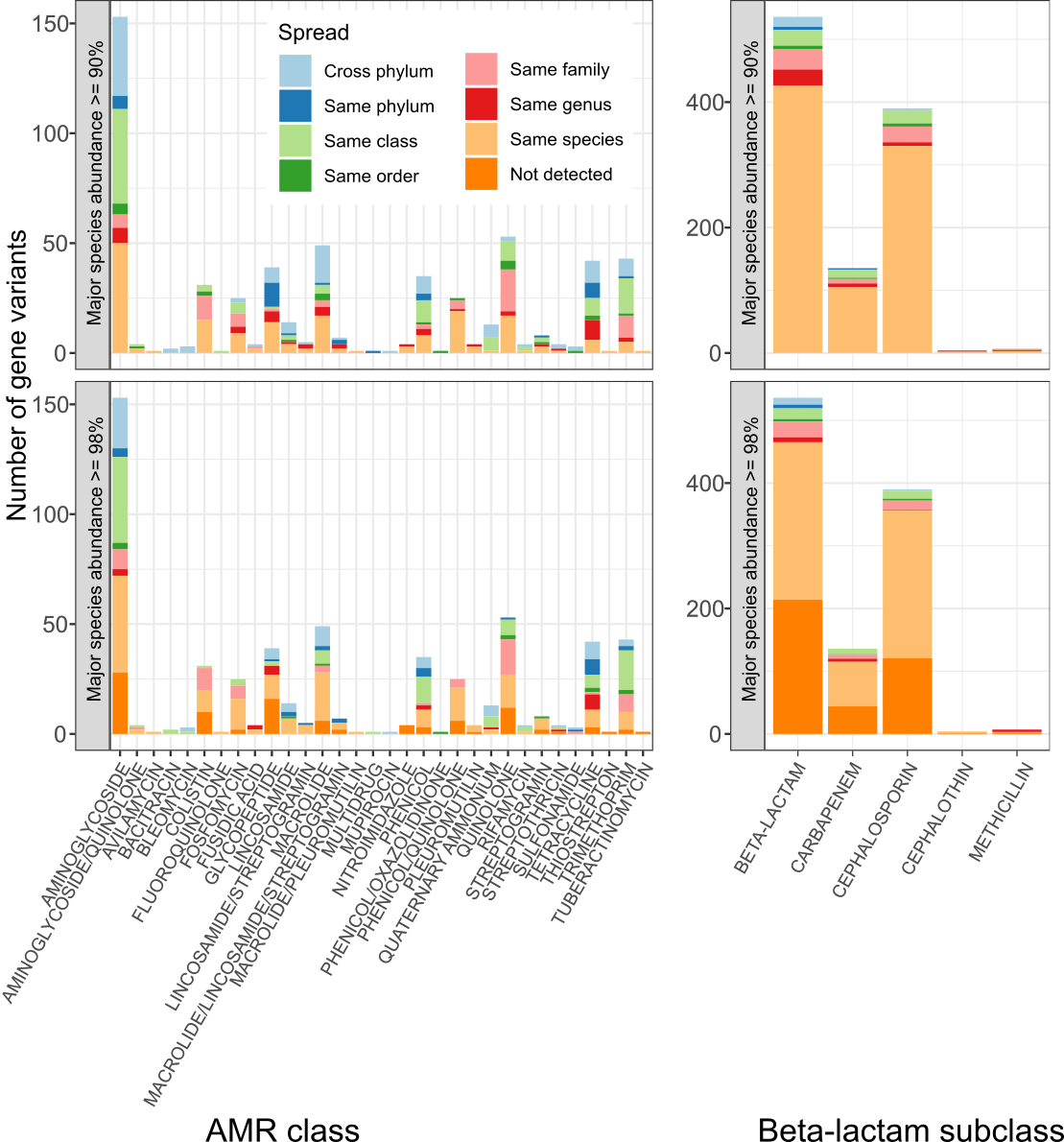

Supplement: S6 Fig — Gene variants are coloured by their level of spread, from being detected in genomes from different phyla to only found in a single species. The top graph includes genomes with the major species being > = 90% abundance, and the lower graph is when this threshold was increased to > = 98% abundance. The data underlying this figure may be found in https://doi.org/10.6084/m9.figshare.16437939. AMR, antimicrobial resistance. (TIFF) [file pbio.3001421.s007.tiff]
