# Supplementary material for: Exploring bacterial diversity via a curated and searchable snapshot of archived DNA sequences
Source: PLoS Biol. 2021 Nov 9;19(11):e3001421. doi: 10.1371/journal.pbio.3001421 (PMC8577725; doi:10.1371/journal.pbio.3001421)
Supplement: Attachment — Submitted filename: Response_to_Reviewers.docx [file pbio.3001421.s008.docx]

**Reviewer #1:** Blackwell et al. describe a curated resource of bacterial genomes that they have generated from ENA records. They process this data using a particular workflow with state-of-the art, appropriate methods and analyse it with respect to taxonomic composition and AMR gene occurrence.

1. Overall, while the technical part is well done, it seems to me that more exciting things and a more-in depth analyses of individual questions might be learned from this data resource than what is presented now in the article.

*We thank the reviewer for their great comments and are glad that they recognise the state-of-the art workflow methods we have used to deliver these data. In developing a resource of this nature, we clearly wanted the emphasis here to be on its value to the community and its content as well as to highlight the limitations inherent in the data that us and others have contributed to the public archives over the last decades. We don’t disagree that there is a lot more that can be done with this resource, but by producing the assemblies and the additional indexes, we believe we have removed many of the barriers that restrict the full use of this data by individuals of different informatic skill levels and computational resource availability. To highlight the increased utility of the 661K resource more clearly, we have added a paragraph in the introduction about analyses and questions that are unable to be easily answered using current public databases. Through the generation and release of this resource we have provided the opportunity for individuals to ask and test their own hypotheses on an unprecedented scale. However, the biases within the data need to be clearly presented so that future conclusions drawn from this data can take these into account.*

2.	As their main conclusion, in the abstract the authors state: Whilst these archives are rich in data, considerable processing is required before biological questions can be addressed…. An analysis on this scale revealed the uneven species composition in the ENA/public databases, with just 20 of the total 2,336 species making up 90% of the genomes. The over-represented species tend to be acute/common human pathogens." That most data in public genome databases comes from very few, cultivable bacteria, most of which are human pathogens is a well-known fact, see, for instance, <https://pubmed.ncbi.nlm.nih.gov/11864374/> ; <https://www.ncbi.nlm.nih.gov/pmc/articles/PMC4361730/>; <https://pubmed.ncbi.nlm.nih.gov/18341921/>

*It’s clear that we knew the principle yes, we agree, but what is also clear is that we had no appreciation of the extent or scale of bias represented in the standing genomic archive, until now. In the Hugenholtz 2002 (267 genomes) and the Land et al 2015 (30,000 genomes) studies they show at a very high level that the top 4 phyla are the same (Proteobacteria, Actinobacteria, Firmicutes and Bacteroidetes) as seen here our study (comprising 661,405 genomes representing 84.19% of all non-redundant submissions to the ENA up until 2018). However, with the difference in scale comes a higher resolution and more certainty. The relative composition is different: Proteobacteria dominate, from 43% or 46% to 60.83% in the 661K snapshot, compared to Hugenholtz et al., and Land et al, respectively. What is more, the lack of overall diversity within our public datasets is getting worse with phyla not represented in the big four decreasing from 29% in 2002, 8% in 2015 and now to 0.68% of all submissions analysed here in the 661K genomes. Thus, our report shows that despite earlier studies we are becoming even more myopic with respect to our understanding of bacterial diversity and the scale, extent or possible ramifications of this we would strongly argue are not widely appreciated and are not laid out in sufficient detail or breadth. Combined with the state-of-the-art standardised analysis this is the reason for this study.*

*To highlight the increasing bias in the public archive we have added the following sentence to the discussion:*

*“The over-representation of species belonging to Proteobacteria, Actinobacteria, Firmicutes or Bacteriodetes phyla is a trend consistent with reports of public database composition from 2002 [39] and 2015 [40]. Concerningly, there has been a reduction in the species diversity outside of these four phyla, decreasing from 29% in 2002, 8% in 2015 and 0.68% of all submissions analysed in the 661K snapshot.”*

3.	Furthermore, I do not think that it is as complicated or time-consuming to come to this conclusion as the authors think using other approaches. One could, for instance, go to the NCBI taxonomy site, select the filter „has genome sequences", display 5 levels and sort the results locally by number of genomes.

*We agree that you can analyse the database composition by using the metadata submitted alongside the genome record. This of course relies on accurate recording at the point of submission. Here we inferred the taxonomic description from the genomic data itself. What is more, and as we describe in the manuscript, by comparing this to the recorded metadata we showed that 8.7% of the species assignments given on submission were incorrect.*

*But of course to discover this you need to process and analyse the data in a very uniform manner and have access to the whole genomic data set. To reinforce the analysis presented and the way we have processed, presented and made accessible these data we have altered the abstract to better highlight the richness, uniformity and quality of our processing, the analytic outputs (AMR, serotype, plasmid rep types etc) and searchability that make what we have presented here unique.*

*The abstract now reads:*

*“The open sharing of genomic data provides an incredibly rich resource for the study of bacterial evolution and function, and even anthropogenic activities such as the widespread use of antimicrobials. However, these data consist of genomes assembled with different tools and levels of quality-checking, and of large volumes of completely unprocessed raw sequence data. In both cases, considerable computational effort is required before biological questions can be addressed. Here, we assembled and characterised 661,405 bacterial genomes retrieved from the European Nucleotide Archive (ENA) in November of 2018 using a uniform standardised approach. Of these, 311,006 did not previously have an assembly. We produced a searchable COBS index, facilitating the easy interrogation of the entire dataset for a specific sequence (e.g. gene, mutation or plasmid). Additional MinHash and pp-sketch indices support genome-wide comparisons and estimations of genomic distance. Combined, this resource will allow data to be easily subset and searched, phylogenetic relationships between genomes to be quickly elucidated, and hypotheses rapidly generated and tested. We believe this combination of uniform processing and variety of search/filter functionalities will make this a resource of very wide utility. In terms of diversity within the data, a breakdown of the 639,981 high quality genomes emphasised the uneven species composition of the ENA/public databases, with just 20 of the total 2,336 species making up 90% of the genomes. The over-represented species tend to be acute/common human pathogens, aligning with research priorities at different levels from individual interests to funding bodies and national and global public health agencies.”*

**Reviewer #2:** The article by Blackwell and colleagues describes a novel resource: a large database of >660k bacterial genomes created from short-read data downloaded from European Nucleotide Archive in 2018. The genomes underwent a consistent assembly pipeline with an extensive quality control and a number of extra post-processing steps, including kmer indexing and minhash sketching. In addition, the authors have provided various analyses aimed at describing the genetic diversity, sequencing trends and the distribution of antimicrobial resistance genetic markers.

1.	I find the article well written, methodology sound and the analyses interesting. Most importantly, the study offers both an exceptional new methodology and a fantastic resource for the entire field of biology. In my opinion the article deserves to be published in PLoS Biology.

*We thank the reviewer for their kind words and recognising the broad appeal and value of this data resource and analysis at a time when it has reached a scale where we need to know both the opportunity to use it to understand deep evolutionary questions around bacterial evolution as well as be entirely cognisant about the inherent biases in this data that may influence our view.*

2.	My major and only reservation is that the article in its current form may leave a reader with an impression that this is yet another bacterial genome database as the authors have not sufficiently explained the pros and cons of using this resource as opposed to another database like NCBI, GTDB or PATRIC. In fact the authors have stated directly that both NCBI and GTDB contain an order of magnitude greater number of bacterial species than the ENA archive. However, it is unclear whether the large number of assemblies provided with this resource is due to oversampling of major epidemiological lineages or due to inclusion of multiple novel lineages of the species already present. The ENA archive could also contain some species which are not present in other databases. To clarify this, I would suggest to include an additional set of analyses using minHash distances at various thresholds to compare what proportion of the genomic space provided by ENA is not covered in other databases like GTDB and PATRIC, and vice versa. A large increase in the number of genomes of major sequence types (eg, ST131 in E. coli or ST258 in Klebsiella) would be a great contribution for many phylodynamic studies which explicitly try to estimate parameters of bacterial evolution, or for the studies of the evolution of bacterial accessory genomes.

*We thank the reviewer for their comments. As you have appreciated above the main contribution of this work is the large set of* ***uniformly processed****, curated and characterised assemblies, but as the reviewer pointed out, ours is not the biggest dataset out there. As suggested, we have compared our 661k dataset with the full set of NCBI assemblies and PATRIC. GTDB was not included as many of its genomes originate from metagenomic samples, not individual isolates like the other databases. By comparing the sample accession (also called biosample) of the assemblies in the 661K, NCBI assemblies and PATRIC databases, the overlap and differences in composition was determined. The upset plot in Supplementary Figure 4A shows that the 661K assemblies has contributed 311,006 assemblies that were not available in GenBank or PATRIC databases. Hence, in addition to providing a large dataset that has been uniformly assembled and which supports multiple search functionalities, we also provide many novel assemblies that were previously absent from other public databases. Supplementary Figure 4B shows the breakdown of the top 50 species in the genomes unique to the 661K and there is a large increase in E. coli and K. pneumoniae genomes, as suggested by the reviewer. A simple summary of the MLST data for these unique assemblies revealed that 3,364 were ST131 E. coli genomes and 1,117 were ST258 K. pneumoniae genomes. These genomes would be valuable for in-depth analyses of these sequence types of interest.*

Minor comments:

1.	Based on Figure 2 there seems to be a correlation between species abundance and the number of AMR genes. Is that driven by the clinical interest (problematic strains are more likely to be sequenced) or by a stronger signal to find AMR with enough genomes?

*We think it is driven by clinical interest, as you suggest.*

2.	It is unclear whether there is any chance of linking non-genetic metadata (eg, ecology or sampling date) with the ENA strains. If not (as I expect), it should be mentioned as one drawback of using a resource like this.

*We were able to link to any additional metadata available in the ENA. These have been collated into an additional file on Figshare.*

3.	I would really love to see a few basic summary plots of the assembly statistics, like the number of contigs, N50, CheckM parameters etc.), as one of the supplementary figures

*Done, see additional supplementary figure (Supplementary Figure 1)*

4.	116-117: this sentence is unclear; not ideal for what exactly, species identification?

*Yes we were referring to species assignment. The relevant sentence has been updated to improve clarity.*

5.	542: I found y-axis title sub-optimal, maybe remove "covered"?

*Done, the y-axis title has been updated in Figure 1B and Supplementary Figure XB to remove “covered”.*

6.	Figure 1: some colours are hard to distinguish (eg, C. coli and C. difficile), I'd suggest changing the colour scale or showing genera, not species.

*Done, the colours have been updated for maximal contrast.*

7.	Figure 2: please mark the most abundant species (say top 20).

*Done, the genera containing members of the 20 most prevalent species in the 661K snapshot are now indicated in bold on the x-axis.*

8.	Also, why Actinobacteria are not shown? Please explain in the legend.

*Actinobacteria were not included in Figure 2 as they did not contain a species on the WHO priority pathogens list and this has now been detailed in the figure legend. However, they were included in Figure 3 as they are one of the 3 orders to contain the MCR species.*
